# Anti-Stress, Behavioural and Magnetoencephalography Effects of an l-Theanine-Based Nutrient Drink: A Randomised, Double-Blind, Placebo-Controlled, Crossover Trial

**DOI:** 10.3390/nu8010053

**Published:** 2016-01-19

**Authors:** David J. White, Suzanne de Klerk, William Woods, Shakuntla Gondalia, Chris Noonan, Andrew B. Scholey

**Affiliations:** 1 Centre for Human Psychopharmacology, Swinburne University of Technology, Hawthorn VIC 3122, Australia; dawhite@swin.edu.au (D.J.W.); sgondalia@swin.edu.au (S.G.); 2 Division of Pharmacology, Utrecht University, 3508 TC Utrecht, The Netherlands; suzanne_dk@hotmail.com; 3 Brain and Psychological Sciences Research Centre, Swinburne University of Technology, Hawthorn VIC 3122, Australia; wwoods@swin.edu.au; 4 HealthGuidance, Inc., Santa Monica, CA 90403-5104, USA; chris@healthguidance.us

**Keywords:** l-theanine, stress, mood, cognition, cortisol, alpha activity

## Abstract

l-theanine (γ-glutamylethylamide) is an amino acid found primarily in the green tea plant. This study explored the effects of an l-theanine-based nutrient drink on mood responses to a cognitive stressor. Additional measures included an assessment of cognitive performance and resting state alpha oscillatory activity using magnetoencephalography (MEG). Thirty-four healthy adults aged 18–40 participated in this double-blind, placebo-controlled, balanced crossover study. The primary outcome measure, subjective stress response to a multitasking cognitive stressor, was significantly reduced one hour after administration of the l-theanine drink when compared to placebo. The salivary cortisol response to the stressor was reduced three hours post-dose following active treatment. No treatment-related cognitive performance changes were observed. Resting state alpha oscillatory activity was significantly greater in posterior MEG sensors after active treatment compared to placebo two hours post-dose; however, this effect was only apparent for those higher in trait anxiety. This change in resting state alpha oscillatory activity was not correlated with the change in subjective stress response or the cortisol response, suggesting further research is required to assess the functional relevance of these treatment-related changes in resting alpha activity. These findings further support the anti-stress effects of l-theanine.

## 1. Introduction

l-theanine (γ-glutamylethylamide) is an amino acid primarily found in the green tea plant (*Camellia sinensis*) and is also present in other species of *Camellia*, as well as in the edible bay boletes mushroom *Xerocomus badius*. The l-theanine content of tea varies considerably, with estimates around 1%–2% of the dry weight of leaves [1,2] and a single cup of tea containing around 25 mg of l-theanine [3]. l-theanine has been shown to cross the blood-brain barrier, reaching peak concentrations in mammals between 30 and 120 min [4,5]. In humans, ingestion of l-theanine doses ranging from approximately 25–100 mg, whether in tea or aqueous solution, result in dose-dependent increases in plasma theanine [6].

Chemically similar to glutamate in structure, l-theanine has also been shown to bind to glutamate receptor subtypes, however with relatively low affinity [7]. Research in lower mammals has reported a complex range of neurochemical actions associated with l-theanine administration, including inhibiting glutamate reuptake [8], increasing brain GABA [9] and striatum dopamine and glycine concentrations [4,10], while serotonin levels were reported to decrease globally, with region-specific increases in the striatum, hippocampus and hypothalamus [4,11]. Longer term l-theanine administration has demonstrated additional actions, including facilitating long-term potentiation and an increase in brain-derived neurotrophic factor (BDNF) expression in the hippocampus over three to four weeks [12,13], as well as mounting evidence supporting a neuroprotective effect [7,14,15].

Due to the fact that l-theanine is traditionally co-consumed with caffeine and other bioactive constituents of tea, a large portion of previous research in humans has studied the combined effects of these ingredients on mood and cognitive function (for reviews, see [16,17,18]). When studied in isolation, the potential anti-stress or anxiolytic effects of acute l-theanine administration have been the predominant focus.

Lu *et al.* [19] used an anticipatory anxiety paradigm to investigate the anxiolytic properties of l-theanine (200 mg) using a placebo-controlled trial plus alprazolam, a conventional benzodiazepine anxiolytic, as an active comparator. While the study found no evidence of a reduction in experimentally-induced anxiety, l-theanine treatment was associated with a significant reduction in baseline anxiety as assessed by the “tranquil-troubled” item of the Bond–Lader visual analogue scales [20]. The authors suggested the experimentally-induced anxiety using electric shock may have been too large to maintain the benefit of the comparatively mild anxiolytic effect of l-theanine, particularly given that the study failed to observe anxiolytic effects for either l-theanine or alprazolam [19]. In contrast, Kimura *et al.* [21] reported significantly reduced subjective stress and anxiety response to a cognitive stressor after receiving 200 mg of l-theanine compared to placebo. This reduction in subjective stress was accompanied by a reduced stress response in secretory immunoglobulin A and heart rate, as well as a trend towards a reduction in the ratio of low frequency to high frequency components of heart rate variability, which taken together were interpreted as evidence that l-theanine administration can reduce the sympathetic nervous system response to acute stress. Performance on the 20-min arithmetic task used as a cognitive stressor was not reported. Yoto and colleagues [22] reported a decrease in subjective stress response to a cognitive stressor after 200 mg l-theanine administration, using the tension-anxiety subscale of the Profile of Mood States [23], but no cognitive performance changes. This study also reported a reduction in blood pressure increases associated with cognitive and physiological stressors (cold pressor test) in the l-theanine condition when compared to placebo. Slightly conflicting results were reported by Higashiyama *et al.* [24], in that 200 mg of l-theanine were not associated with subjective anxiety reductions using a placebo-controlled cross-over design, while reporting a reduction in heart rate and improvements in cognitive performance during completion of visual attention and auditory oddball tasks. These effects were only apparent in a higher trait anxiety subgroup.

A number of studies have systematically explored the isolated and combined effects of l-theanine and caffeine on mood and cognitive performance, typically observing cognitive enhancing effects for combined treatment, but little cognitive benefit for l-theanine alone. Rogers *et al.* [25] found that a 200-mg l-theanine treatment in isolation was not associated with changes in mood and was found to increase reaction times on a visual probe task designed to detect bias towards threat-related words (in the case of faster response times). The study did however find that the combined l-theanine and caffeine treatment protected against the increase in blood pressure associated with caffeine administration (250 mg). Haskell *et al.* [26] also studied l-theanine and caffeine in isolation and combination and found little evidence in support of cognitive or mood effects of l-theanine in isolation. The combined treatment was associated with a number of performance improvements, including simple reaction time, rapid visual information processing (RVIP) accuracy and numeric working memory response times. l-theanine (250 mg) alone was associated with elevated headache reports and impaired serial subtraction performance 90 min post-dose. Haskell *et al.* did however note that follow-up contrasts showed that l-theanine was associated with significantly greater subjective calmness assessed by the Visual Analogue Scale 30 min post-dose when compared to placebo.

Changes in brain alpha oscillatory activity using electroencephalography (EEG) have also been studied as correlates of l-theanine’s potential anti-stress effects, using resting state recordings, and cognitive enhancing effects, using task-related recordings. The first study to report changes in human brain activity associated with l-theanine administration described changes in alpha oscillatory activity (8–13 Hz) during a resting state in scalp electrodes over parietal and occipital regions [27,28]. This study explored changes in alpha activity within 60 min of administration of water or 50-mg and 200-mg doses of l-theanine in female participants, four high and four low in trait anxiety, reporting a dose-dependent increase in alpha power with trending towards significance across all participants, but significantly greater in the high anxiety group. Similarly, Higashiyama and colleagues [24] reported significantly elevated alpha activity during rest periods between cognitive tasks in the 60 min following 200-mg l-theanine administration compared to placebo; again, this effect was only apparent for eight participants showing higher trait anxiety. This study failed to observe a concomitant change in subjective anxiety associated with this effect on alpha oscillatory activity. Significantly increased alpha activity has also been described 45–105 min after up to 50 mg of l-theanine, consumed as part of a tea infusion, in a parallel group design compared to placebo in healthy participants [29], as well as in high anxiety participants one hour after administration [30]. These findings are typically interpreted in line with the proposed relaxant properties of l-theanine when recordings are taken at rest; however, while research has linked lower resting state alpha activity with states of autonomic arousal associated with caffeine [31,32], no study to date has explored the association between these changes in resting state alpha activity and potential anti-stress benefits of l-theanine administration. This is particularly important, as the complex functional role of resting alpha oscillatory activity is still emerging [33], and resting state alpha activity can be considered at best a crude indicator of an individuals’ state of stress, anxiety or relaxation [19]. Beyond these changes in resting alpha oscillatory activity, studies to explore functional changes in brain oscillatory activity associated with l-theanine activity during cognitive task engagement have reported tonic shifts towards reduced alpha activity during attention-demanding tasks with possible phasic alpha increases facilitating the inhibition of task irrelevance, suggesting additional actions beyond those observed in a resting state [34,35,36]; however, these functional changes remain to be fully characterised, particularly given the limited evidence of behavioural performance improvements on cognitive tasks associated with l-theanine administration in humans [25,26,35].

Thus, research to date suggests a potential anti-stress effect of a single dose of l-theanine in humans, with limited support for cognitive enhancing effect based on behavioural studies in humans when consumed without caffeine. Further, there is neurophysiological evidence of a shift in resting alpha oscillatory activity after l-theanine administration, which is often interpreted in support of the potential anti-stress effects of l-theanine; however, no study to date has simultaneously reported subjective stress and resting alpha oscillatory activity changes in response to l-theanine treatment.

Based on these potential benefits to mood and general health, l-theanine is emerging as an ingredient of interest for functional foods and drinks, either synthesised or extracted from green tea [37]. The present study reports on a randomised, placebo-controlled, double-blind, crossover study investigating the anti-stress, cognitive and neurophysiological effects of an l-theanine-based nutrient drink. The l-theanine-based active treatment utilised in the present study was a nutrient drink, commercially available as NeuroBliss^®^. This nutrient drink contains l-theanine (200 mg), in addition to smaller amounts of phosphatidylserine (PS; 1 mg), alpha glycerylphosphorylcholine (GPC; 25 mg) and chamomile (10 mg). Beyond the l-theanine content, previous research has typically explored PS, alpha GPC and chamomile at much higher doses and as part of chronic dosing schemes. For example, preliminary evidence suggests chronic supplementation with phospholipids at high doses may prove beneficial in treating a range of diseases and enhancing brain function [38]. Of particular relevance to the present research, daily supplementation with 300–400 mg of PS over three to four weeks has been found to reduce the stress response to laboratory-induced mild stress [39,40]. A randomised controlled trial of chamomile extract (*Matricaria recutita*) supplementation over eight weeks in generalised anxiety disorder also suggests anxiolytic effects of this extract [41]. Thus, while the primary active constituent under investigation in the present study was l-theanine, which has demonstrated acute effects and is contained in high amounts, the outcomes of the study must be considered in light of the presence of these small doses of PS, alpha GPC and chamomile within the nutrient drink.

## 2. Methods

This study used a randomised, placebo-controlled, double-blind crossover design to evaluate the anti-stress, cognitive and neurophysiological effects of an l-theanine-based nutrient drink. The study was approved by the Swinburne University Human Research Ethics Committee (SUHREC Project ID 2013/073) and was registered with the Australian New Zealand Clinical Trials Registry (ACTRN12614000826640).

### 2.1. Participants

Thirty-six healthy adults aged 18–40 were enrolled in the study, with 34 completing all three study visits (one participant lost to follow-up and one protocol violation). Criteria for inclusion required participants be non-smokers, free of significant concurrent illness, including diabetes mellitus, bleeding disorders, heart conditions and psychiatric diagnosis. In addition, the presence of health conditions with the potential to affect food metabolism, for example food allergies, kidney or liver disease, were considered criteria for exclusion, as well as a history of head injury, epilepsy or stroke. Demographic information for participants is provided in Table 1 below.

**Table 1:** Demographic information for study participants.

Variable		Full Sample (*n* = 34)	Subset MEG Participants (*n* = 17)
Gender	Male	15	8
	Female	19	9
Age (years)	Mean	26.53	27.03
	SD	(5.04)	(5.61)
BMI (kg/m^2^)	Mean	22.66	22.99
	SD	(2.86)	(2.86)
Trait Anxiety	Mean	37.56	37.76
(STAI-T)	SD	(7.44)	(6.95)
Years of Education	Mean SD	17.25 (2.85)	17.74 (3.10)

BMI, Body mass Index; STAI-T, Spielberger State-Trait Anxiety Inventory—Trait portion; MEG, magnetoencephalography.

### 2.2. Procedure

Participants attended the Centre for Human Psychopharmacology on three occasions; one screening/practice day and two counterbalanced experimental testing days. A staff member of the Centre for Human Psychopharmacology external to the trial was responsible for allocating treatment sequences, with block randomisation used to ensure treatment sequences were balanced across the sample of 36 participants. A minimum 48-h washout period was required between full testing days, with the maximum gap between testing visits being 14 days. Participants were required to abstain from alcohol in the 24 h preceding each testing visit and caffeine in the 12 h prior to testing.

At the initial screening visit, voluntary written informed consent was obtained from participants prior to any study procedures being performed. Subsequently, this visit involved full assessment of eligibility, obtaining basic morphometric and demographic information and medical history, in addition to obtaining baseline measures of mood and anxiety. Finally, participants were familiarised with cognitive and mood assessments to be administered during the two full testing sessions.

The two full testing days followed identical procedures, in which participants completed a cognitive stressor on three occasions (pre-treatment baseline, then one and three hours post-dose). Time of day was kept constant, with participants receiving a standardised light meal upon arrival at the laboratory (11:00), baseline stressor assessment beginning late morning (11:30) and the dose being given at midday (12:00). Measures of stress, fatigue and mood in addition to salivary cortisol were obtained immediately prior to and following each completion of the cognitive stressor. A subset (*n* = 17) of participants also participated in magnetoencephalography (MEG) recordings two hours post-dose in order to assess potential neurophysiological changes associated with treatment. l-theanine has been shown to reach peak concentrations in mammals between 30 and 120 min [4,5], with peak plasma concentrations in humans reported approximately 50 min post-dose [6]. Evidence from lower mammal research suggests that brain l-theanine levels may be increased after one hour, but peak as much as five hours post administration [42]. Thus, assessment one and three hours post-dose captures a period in which plasma and brain l-theanine concentrations are significantly elevated.

### 2.3. Treatments

Participants consumed both interventions orally as a drink of approximately 430 mL (14.5 fluid ounce). The l-theanine-based active treatment utilised in the present study was a nutrient drink, commercially available as NeuroBliss^®^. The active treatment contained l-theanine (200 mg, l-Tea-Active^®^; Blue California, CA, USA), l-alpha glycerylphosphorylcholine (alpha GPC; 25 mg), phosphatidylserine (1 mg) and micronized chamomile (10 mg). Both active and vehicle control treatments contained identical sweeteners (crystalline fructose and sucralose), preservatives (sodium benzoate, potassium sorbate), gum acacia and malic acid.

#### Analysis of Theanine Content

Independent assessment confirmed the l-theanine content of the active (197 mg l-theanine; <1 mg d-theanine) and placebo (<1 mg l-theanine; <1 mg d-theanine) treatments using high-performance liquid chromatography (HPLC; Eurofins Scientific Inc., Petaluma, CA, USA). Further detailed analysis of the enantiomeric composition of the theanine content confirmed the predominance of the l-isomer in both isolated l-Tea-Active (≤0.2% d-theanine) and the combined active treatment used in the study (0.2%–0.3% d-theanine) using an HPLC-mass spectrometry method previously used for theanine isomer analysis [43].

### 2.4. Cognitive Stressor: Multi-Tasking Framework

This study used the multi-tasking framework (MTF; Purple Research Solutions) to act as both a stressor and an assessment of cognitive performance [44]. This task was completed three times at each assessment visit: at pre-treatment baseline, then one and three hours post-dose. As part of the MTF, four tasks were displayed, one in each quadrant of the monitor, which were required to be completed simultaneously for a period of 20 min for each of the three assessments at each testing day. Completion of the MTF has previously been shown to increase both subjective stress and physiological measures, such as heart rate and blood pressure [44]. The implementation of the MTF used in the current study was set to medium intensity and was identical to that described in previous investigations of anti-stress and cognitive enhancing properties of glucose and caffeine [45], chewing gum [46], *Bacopa monnieri* [47] and multi-vitamin supplementation [48].

An overall score is displayed in the centre of the screen, which reflects the speed and accuracy of performance across the four tasks. Briefly, the four tasks used in the MTF were mathematical processing, Stroop (colour-word), memory search and psychomotor tracking. Mathematical processing required participants to complete the addition of two three-figure numbers, entering their response using a number pad on the screen. The Stroop task presented colour words (“red”, “yellow”, “green” and “blue”) in text colour of one of the four corresponding colours one at a time, with participants required to indicate the font colour by clicking on coloured panels within 20 s. The memory search task presented an initial memory set of four letters, which disappears after four seconds; then single probe letters were presented, and participants had 15 s to indicate whether each probe belonged to the initial memory set by clicking a corresponding button (true or false). Psychomotor tracking presented an outwardly moving red dot, within a target-shaped series of concentric circles. Participants were required to press a “reset” button before the red dot moved beyond the outermost circle. Scoring for mathematical processing, Stroop and memory search tasks involved +10 points for correct responses and −10 points for incorrect responses or timeouts. Scoring for psychomotor tracking involved a maximum of +10 points for resetting in the outermost circle to +2 in the innermost and losing points at a rate of −10 points for every 0.5 s where the dot reached the edge of the outermost circle without being reset. The combined overall score, in addition to scores on the four individual tasks, formed the cognitive performance outcomes in the present study.

### 2.5. Assessment of Stress, Mood and Fatigue

Stress, mood, fatigue and salivary cortisol were assessed immediately prior to and following each completion of the MTF. The primary outcome measure, stress, in addition to other measures of mood and fatigue were assessed before and after each completion of the MTF using a series of visual analogue mood scales, as well as the “State” subscale of the State-Trait Anxiety Index (STAI-S; [49]). Visual analogue mood scales have been shown to possess high reliability and validity [50].

#### 2.5.1. Bond–Lader Visual Analogue Mood Scales

The Bond and Lader [20] mood scales have been used extensively in studying the mood effects of a range of psychopharmacological interventions, and changes as a result of MTF completion have been demonstrated [44]. The scale consists of 16 items, each a 100-mm line with antonyms at either end (for example, “lethargic” and “energetic”). Three subscales are derived from these responses, as the average distance (millimetres) from the negative antonym of each contributing item, providing a measure of alertness, calmness and contentedness.

#### 2.5.2. Stress and Fatigue Visual Analogue Mood Scales

Two additional measures of subjective mood, stress and fatigue, were administered in the form of visual analogue scales. These items asked participants to rate “How stressed/fatigued do you feel right now?” with each item having a 100-mm line and the words “extremely” and “not at all” at either end. Items were scored as distance from the low end, such that higher scores represent more stress or fatigue.

#### 2.5.3. State-Trait Anxiety Inventory: State Subscale

The STAI-S [49] is a widely-used, self-rated measure of anxiety in the present emotional state, with strong psychometric properties and sensitivity across a broad range of experimental paradigms testing state-dependent fluctuations in anxiety (for a review, see [51]). The STAI-S contains 20 items, each containing a short statement to which current intensity (“right now, at this moment”) is rated on a 4-point Likert scale (e.g., “I feel nervous”, 1 = not at all, 4 = very much so). Scores range from 20–80, with higher scores representing higher state anxiety. In addition, the Trait subscale was administered at the screening visit.

### 2.6. Salivary Cortisol

Saliva samples were collected immediately prior to and following each completion of the MTF. Participants were given a Salivette^®^, which they were instructed to put in their mouth, either chewing or placing under the tongue, allowing saliva to soak the swab for a minimum of 30 s. They then put the swab into a labelled container, and it was stored in a freezer at −20 °C until the completion of the study. Saliva sampling was done before and after each completion of the MTF, resulting in 6 samples per participant at each treatment visit.

Once all saliva samples for the study were collected, they were analysed for levels of cortisol using enzyme-linked immunosorbent assay kits (ELISA). All samples were thawed and analysed using high sensitivity Salivary Cortisol EIA kits (Salimetrics, State College, PA, USA), with intra- and inter-CVs being less than 10%. Samples from four participants were insufficient to obtain salivary cortisol estimates at multiple time points; thus, cortisol analysis proceeded with these four participants excluded. Typically cortisol release peaks immediately following awakening, increasing by up to 50%–70% within the first 30 min, declining throughout the day, more steeply in the morning before somewhat plateauing in the afternoon [52,53]. Testing sessions were conducted at a consistent time of day, starting at 11:30 a.m., in order to minimise the impact of diurnal fluctuations in cortisol. However, this baseline assessment being conducted from 11:30 a.m. suggests any stressor-induced changes in cortisol would be superimposed on declining levels naturally occurring as part of the diurnal cycle. In addition, as participants received a light standardized meal 30 min prior to baseline testing, it is likely that this assessment also captured meal-induced cortisol stimulation.

### 2.7. MEG Subset

A subset of 17 participants underwent an investigation of brain activity during a relaxed resting state at both treatment visits. MEG data were acquired at Swinburne University of Technology, Brain and Psychological Sciences Research Centre, using a 306-channel Elekta Neuromag TRIUX system. The system uses 102 magnetometers and 204 planar gradiometers. Data were acquired with a sampling rate of 1000 Hz, with a single bipolar vertical EOG channel, as well as an ECG channel simultaneously acquired. Participants were in a seated position and instructed to relax and close their eyes, whilst a five-minute resting state recording was obtained. Recordings were conducted between the two post-dose stressor assessments, with recordings beginning approximately 120 min post-dose. In addition, recordings were made during completion of an intersensory cued attention task, the results of which will be reported elsewhere.

### 2.8. Data Analysis: Data Screening and MTF Stressor Responders

Blinded data screening was conducted on all measures. From this data screening, one participant was missing data for the primary outcome measure, and two participants were consistently identified as outliers through a pattern of extreme responses on mood scales and, thus, were excluded from behavioural analyses, leaving 31 participants for analysis of behavioural outcomes.

Prior to unblinding for analysis of treatment effects, a preliminary exploration of the primary outcome variable, the MTF stressor reactivity using the stress visual analogue scale, for the baseline (pre-dose) assessments of both treatment visits was conducted. While the MTF significantly increased stress at baseline for both treatment visits across the sample, those with atypical responses to the stressor at either baseline assessment were excluded from treatment analysis. Five participants showing a decrease of greater than 15 points in the stress visual analogue scale at the baseline assessment for either treatment visit from pre- to post-stressor were excluded from subsequent analysis. This resulted in a “stressor responder” sample of 26 participants (9 male/17 female), mean age 25.79 (SD = 4.74), all of which showed an increase in stress in response to performing the MTF during at least one baseline assessment. This subset was fully balanced for treatment sequence (*i.e.*, 13 received active treatment at the first visit).

### 2.9. Data Analysis: Mood, Cognitive Performance and Cortisol

In order to probe the treatment-related changes in stress and mood response to the cognitive stressor, change from before to after the MTF stressor was computed for each completion of the framework (e.g., Δ = Post Stress-Pre Stress, such that positive scores indicated an increase in the outcome measure from pre- to post-stressor). For each treatment visit, the mood change at the baseline assessment was subtracted from the corresponding mood change for each post-dose assessment for that treatment visit (Δ Δ = Post-doseΔ − BaselineΔ, such that higher scores indicate a greater mood change post-dose). This process follows previous research using the MTF and other acute psychological stressors, adjusting for any day-to-day fluctuations in response to the stressor [46,47,54,55,56]. Similarly, treatment-related changes in cognitive outcomes associated with the multitasking framework were assessed using change in performance from pre-treatment baseline. This was achieved by computing change for each post-dose completion of the framework (e.g., Δ = Post-dose performance − Baseline performance, such that positive scores indicated improved performance after treatment). The change in cortisol from pre- to post-stressor was analysed without adjusting for the baseline change. This was motivated by the fact that the baseline assessment did not show reliable cortisol changes associated with the completion of the stressor, in addition to evidence of considerably higher cortisol at the baseline assessment, suggesting these assessments predominantly captured varying stages of the steeper decline following the awakening response peak, plus meal-induced cortisol stimulation, as participants received a light standardized meal 30 min prior to baseline testing.

Both post-dose time points were entered into a two-way treatment (active, placebo) × time point (1 h, 3 h post-dose) repeated measures ANOVA for each outcome. Where significant main effects or interactions were observed, pairwise comparisons were used to assess each time point at a Bonferroni adjusted threshold (α = 0.025). Where there was evidence of violations to the normality assumption, transformation of data was first attempted. Further violations of the assumptions of the parametric test, or in the event that transformation was unsuccessful, the non-parametric Wilcoxon signed rank test for matched pairs was used at each post-dose time point separately.

### 2.10. Data Analysis: MEG Data

Pre-processing of MEG resting state data involved first removing environmental noise using the temporal signal space separation (tSSS) method implemented in MaxFilter Software [57]. Subsequently, data were processed in MATLAB 2014a (Mathworks Inc., Natick, MA, USA), using both Brainstorm software [58] and custom scripts. Cardiac artefacts were identified from a single bipolar ECG channel and subsequently removed using signal space projection (SSP) methods [59], as implemented in the Brainstorm software package. Three participants failed to show a well-characterised projection consistent with cardiac artefact, and thus, SSP reduction of the cardiac artefact was not applied to the data from either treatment visit in these cases. All magnetometer data were then visually inspected, with segments containing residual artefacts excluded from subsequent analysis. A minimum of 3 min of accepted data were retained for all sessions, and the two treatment conditions did not significantly differ in mean time retained per session (placebo = 270 s; active = 262 s).

Spectral power at each magnetometer was calculated using Welch’s method, using 4-s epochs with 50% overlap (Hamming windowed). Relative alpha power was calculated by dividing power within the 8–13 Hz band by total power (1–40 Hz). This step was necessary, as absolute power using MEG sensors across sessions is influenced by any differences in head position within the MEG helmet, complicating comparisons across sessions [60].

Mean relative alpha power at each magnetometer site was then contrasted between active and placebo treatment visits. This analysis utilised a permutation test based on the paired samples *t*-statistic contrasting relative alpha power between treatment visits [61,62]. Using the *t*-max method of Blair and Karniski [61], the *p*-values of each sensor site are adjusted for multiple comparisons. As such, this method controls the family-wise error rate (here, *p* < 0.05, one-tailed), like Bonferroni correction; however, the permutation method remains more powerful where variables are correlated (such as that observed in sensors with close proximity).

## 3. Results

### 3.1. Primary Outcome: Subjective Stress Response to MTF

**Figure 1:**
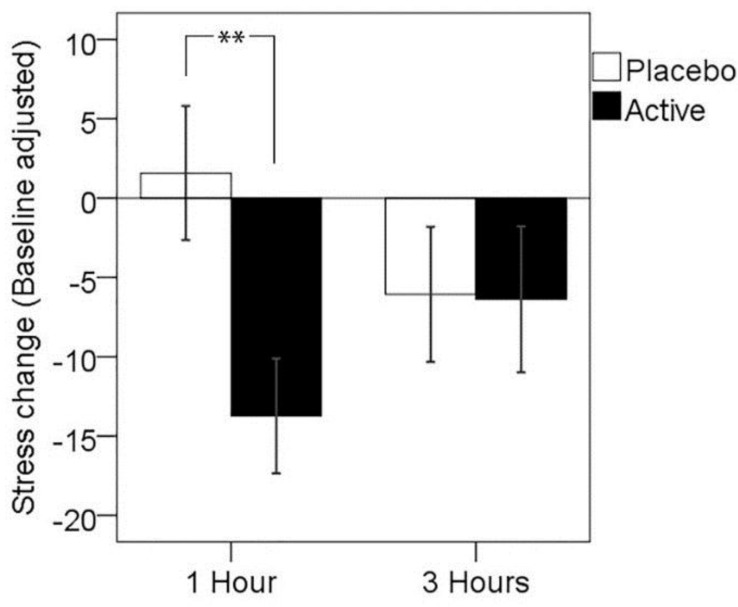
Mean change in stress response, adjusted for baseline, at 1 and 3 h post-dose for each treatment visit. Error bars ± 1 SE; ** *p* < 0.01.

A two-way analysis of variance with treatment visit (placebo, active) and time post-dose (one hour, three hours) as repeated measures was conducted in order to assess the presence of any significant treatment effects in the self-rated stress response to the completion of the MTF. The main effect of treatment was not significant (*F*(1,25) = 2.63, *p* = 0.117, partial *η*^2^ = 0.095), with no main effect of time post-dose (*F*(1,25) < 0.01., *p* > 0.05); however, a significant assessment time by treatment interaction was observed (*F*(1,25) = 6.50, *p* = 0.017, partial *η*^2^ = 0.206). Paired comparisons showed significantly reduced subjective stress response 1 h post-dose for the active treatment when compared to placebo treatment (*p* = 0.003), but no significant difference between treatments in stress response at 3 h post-dose (*p* > 0.05). The mean change in the self-rated stress response is shown in Table 2 below and in Figure 1.

**Table 2:** Baseline adjusted stress response (ΔΔ stress, mean and standard deviation) to the multi-tasking framework (MTF) at both post-dose assessments for placebo and active treatments.

		1 h		3 h	
	N	M	(SD)	M	(SD)
**Placebo**	26	1.58	(21.54)	−6.08	(21.70)
**Active**	26	−13.73	(18.49)	−6.38	(23.48)
****		**		N.S.	

** *p* < 0.01, N.S., *p* > 0.05; pairwise comparisons.

Follow-up analyses were conducted in order to confirm that this effect was not driven by differences in pre-treatment baseline subjective stress response, nor differences in self-reported stress levels prior to MTF completion. Contrasting change in self-rated stress (Δ stress for each post-dose assessment, without baseline adjustment) showed significantly reduced stress response 1 h post-dose for the active treatment when compared to the placebo treatment (*T*(25) = 3.03, *p* = 0*.*006), but no significant difference between treatments in stress response at 3 h post-dose (*p* > 0.05). Paired samples *t*-tests were conducted between treatment visits on self-reported stress pre-MTF and post-MTF for the 1 h post-dose assessment point; this showed no significant difference between pre-MTF stress levels (*T*(25) = −1.31, *p* = 0.202) and a trend towards significantly lower stress levels post-MTF for the active treatment (*T*(25) = 1.71, *p* = 0.099). A follow-up analysis including the treatment sequence as a between subjects factor showed no main effect (*F*(1,24) = 1.58, *p* = 0.222), nor a sequence by treatment by time point interaction (*F*(1,24) = 1.13, *p* = 0.299). These results suggest that the significant effect of treatment on stress was specifically related to a reduced stress response to the completion of the cognitive stressor and not a result of taking into account baseline stress response, nor a result of changes to general mood state or carry-over effects of the treatment sequence.

### 3.2. Mood and Fatigue Response to MTF

While active treatment was associated with small mean reductions in fatigue and anxiety and mean increases in alertness and calmness in response to the MTF, analyses showed no significant main effects of treatment or treatment by time point interactions for these mood measures (Table 3).

**Table 3:** Baseline-adjusted mood response to the multitasking framework 1 and 3 h post-dose for both treatments (Mean (M) and SD), with the *p*-value of the treatment main effect and the treatment by time interaction. STAI-S, State-Trait Anxiety Index.

		1 h		3 h		Treatment × Assessment Time *p*-Values	
		Placebo Active		Placebo Active			
	*n*	M	M	M	M	Treatment	Interaction
		(SD)	(SD)	(SD)	(SD)		
Fatigue	25	−3.32	−5.96	−4.80	−7.44	0.663	0.999
		(23.63)	(22.48)	(23.99)	(23.62)		
Alertness	26	−0.72	3.86	5.28	5.69	0.375	0.267
		(14.19)	(15.44)	(11.77)	(11.30)		
Contentedness	26	−0.56	1.41	1.99	0.62	0.885	0.152
		(7.90)	(7.81)	(9.29)	(7.89)		
Calmness	26	−0.31	3.87	0.25	0.04	0.624	0.418
		(18.39)	(21.36)	(13.61)	(14.64)		
Anxiety (STAI-S)	26	1.50	−1.81	0.12	−1.00	0.201	0.251
		(7.12)	(6.20)	(5.88)	(6.36)		

#### “Tranquil-Troubled” Rating at Rest

An additional analysis was conducted in order to pursue the findings of Lu *et al.* [19], who reported significant relaxant effects of 200 mg l-theanine as measured by the “tranquil-troubled” item of the Bond–Lader visual analogue scales when administered in a rested state. Pre-stressor ratings on this item, as the change from pre-treatment baseline rating, were compared between treatment visits both 1 and 3 h post-dose. Two-way analysis of variance showed no significant main effect of treatment (*F*(1,25) = 0.57, *p* *>* 0.05), nor a treatment × assessment time interaction (*F*(1,25) = 0.59, *p* > 0.05). These ratings are summarised in Table 4.

**Table 4:** Ratings on the “tranquil-troubled” item of the Bond–Lader mood scale, as the change from baseline, at both pre-stressor assessments after the dose for placebo and active treatments (Mean (M) and SD).

		1 h		3 h	
	*n*	M	(SD)	M	(SD)
**Placebo**	26	4.23	(13.61)	2.54	(11.83)
**Active**	26	0.69	(9.69)	1.50	(15.19)

### 3.3. Cortisol Response to MTF

**Figure 2:**
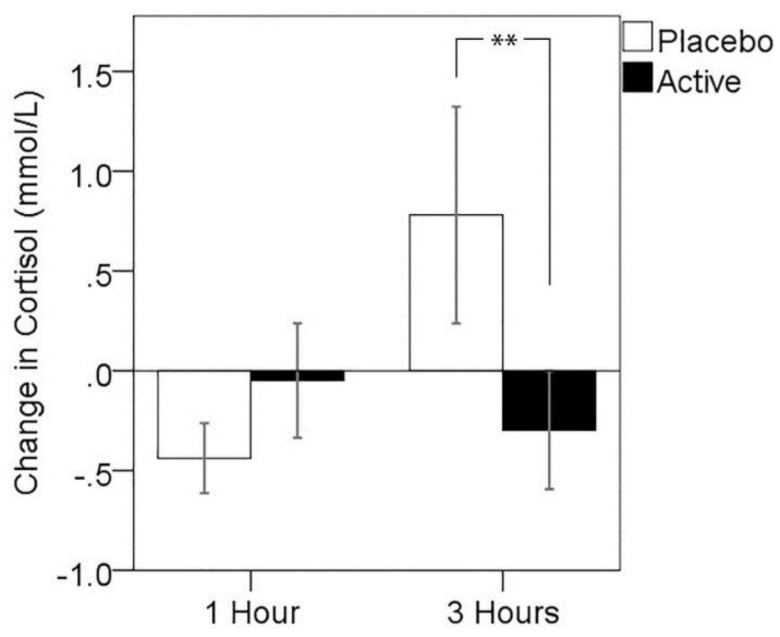
Change in cortisol from pre- to post-stressor for both post-dose assessments and treatment visits. Error bars ± 1 SE, * *p* < 0.05 using the Wilcoxon signed ranks test.

Due to evidence of violations to the assumption of normality in cortisol change, which were not corrected by transformation, non-parametric analysis of treatment effects was conducted. Results of Wilcoxon signed ranks tests showed no significant differences in cortisol change between active treatment compared to placebo 1 h post-dose (*z* = 1.28, *p* > 0.05, median cortisol response: placebo = −0.19, active = −0.02), but a significantly lower cortisol response 3 h post-dose for the active treatment visit (*z* = −1.98, *p* = 0.047, median cortisol response: placebo = 0.44, active = −0.09). A series of analyses were run in order to exclude the possibility that completion of the MEG component of the study immediately preceding this assessment differentially impacted cortisol responses. Mann–Whitney U-tests indicated that cortisol levels did not differ between MEG and non-MEG participants at the 3-h pre-MTF assessment at either treatment visit (placebo: *z* = −0.92, *p* = 0.372; active: *z* = −1.32, *p* = 0.197), nor did the MTF-related change in cortisol differ between MEG and non-MEG participants at either treatment visit (placebo: *z* = −0.92, *p* = 0.372; active: *z* = −0.33, *p* = 0.771) Change in cortisol for each assessment point is plotted for both treatment visits in Figure 2.

### 3.4. Cognitive Performance on MTF

Aside from the overall score change on the MTF, the change in performance for each of the modules was not consistently normally distributed. Thus, two-way analysis of variance with treatment and post-dose time point as repeated measures was conducted for the overall score change, with non-parametric Wilcoxon signed ranks tests conducted for performance change scores on the individual modules of the MTF at each post-dose time point.

**Figure 3:**
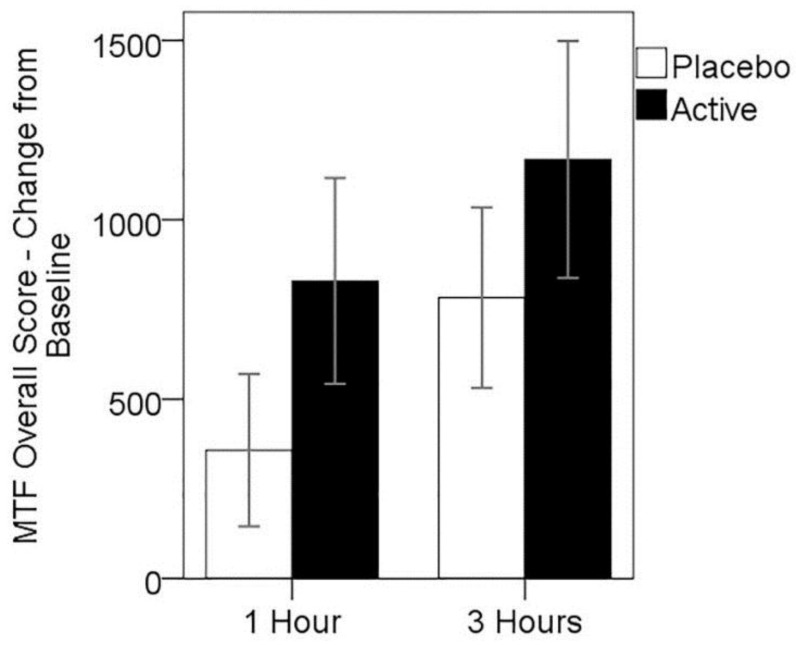
Mean change in MTF overall score from baseline, for both treatments at 1 and 3 h post-dose. Error bars ± 1 SE.

While mean change in overall score from baseline MTF performance was higher for the active treatment at both post-dose assessments (see Figure 3), these differences were not significant. Two-way repeated measures ANOVA showed no significant main effect of treatment (*F*(1,24) = 1.12, *p* > 0.05), nor treatment × assessment time point interaction (*F*(1,24) = 0.07, *p* > 0.05); however, a significant main effect of assessment time point (*F*(1,24) = 6.66, *p* = 0.016), suggesting practice effects were evident over the testing day.

Non-parametric Wilcoxon signed ranks tests showed no significant differences between treatments on performance change for score measures obtained from the four individual modules of the MTF at either post-dose time point.

### 3.5. MEG Resting State Alpha Activity

**Figure 4:**
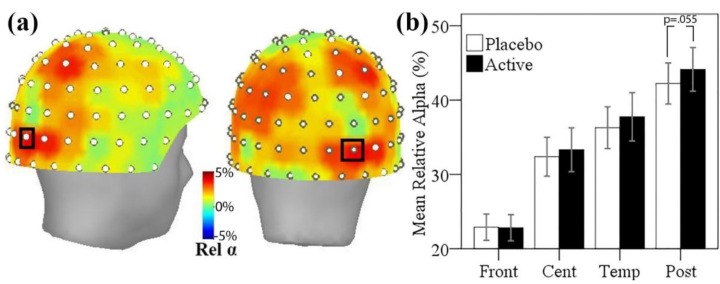
(**a**) Topographic maps of mean relative alpha difference between active and placebo visits viewed from the right side and posterior views (positive numbers = greater at active visit). The sensor showing a trend of *p* < 0.1 is marked with black rectangle; (**b**) Mean relative alpha power at each treatment visit, averaged across magnetometer sensors within frontal (Front), central (Cent), temporal (Temp), and parieto-occipital (Post) regions. Error bars ± 1 SE.

The mean difference in relative alpha power between active and placebo treatment visits is shown in Figure 4a below. A broad trend, maximal in posterior regions, towards greater resting relative alpha power after active treatment, was observed. Uncorrected *p*-values showed five sensor sites with greater alpha power in the active treatment (*p* < 0.05); however, the results of the permutation paired *t*-tests showed no single sensor site with significantly greater relative alpha power using family-wise error correction. A single sensor site over right parieto-occipital regions showed a non-significant trend approaching significance (*p* < 0.1, Familywise error (FWE) corrected), where alpha power comprised 5% more of the total power after the active treatment.

In order to explore more broad treatment-related differences in regional alpha activity, relative alpha power was also studied across sensor regions, with mean relative alpha calculated for frontal, central, temporal and parieto-occipital (posterior) sensor regions. Testing mean relative alpha in posterior regions between placebo and active visits showed a non-significant trend towards greater posterior alpha activity after active treatment (*t*(16) = 1.68, *p* = 0.055, one-tailed). The mean relative alpha within each sensor region is shown for the two treatment visits in Figure 4b.

#### 3.5.1. Resting State Alpha Oscillatory Activity Changes and Trait Anxiety

**Figure 5:**
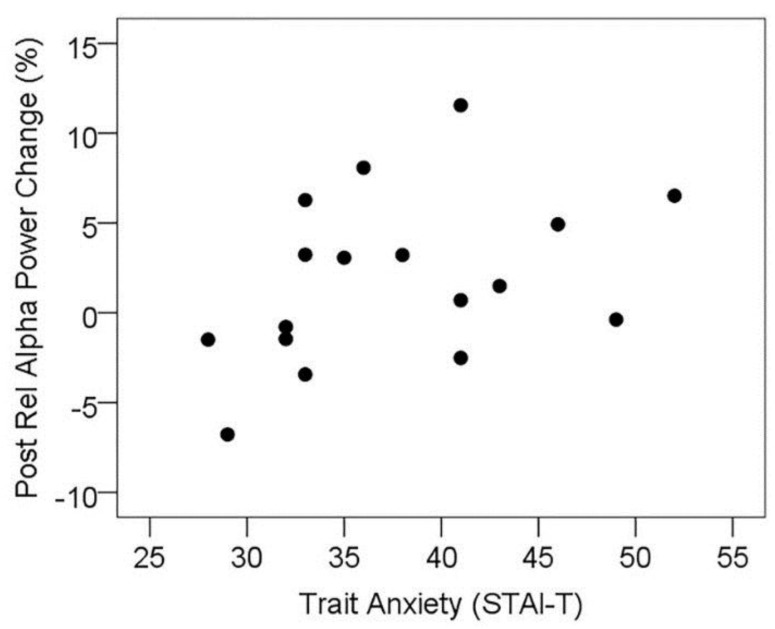
Scatterplot showing the positive association between trait anxiety and treatment-related change in resting relative alpha power in posterior sensor sites (positive change = increased alpha during active treatment visit).

In order to explore the relationship between trait anxiety and the effect of l-theanine administration on resting alpha activity, the change in mean resting alpha activity across posterior sensors from placebo to active visit was correlated with trait anxiety scores from the STAI-T. In agreement with previous research, the change in posterior resting alpha was significantly correlated with trait anxiety scores (*r_s_* = 0.477, *p* = 0.026, one-tailed; see Figure 5). In order to explore this further, the MEG subset of participants was divided into high and low trait anxiety subgroups by median split (STAI-T cut-off 36), and resting posterior alpha activity at the placebo visit was contrasted between high and low anxiety groups; however, these groups did not significantly differ (*t*(15) = 0.29, *p* > 0.1). Within these subgroups, posterior resting alpha was significantly higher at the active treatment visit contrasted with placebo for the high trait anxiety group (*t*(8) = 2.51, *p* = 0.019, one-tailed), while there was no significant change in the low anxiety group (*t*(7) = −0.12, *p* > 0.1), see Table 5. This suggests that the treatment-related increase in resting alpha does not reflect a mechanism of “normalising” alpha in high anxiety individuals, instead selectively enhancing the predominance of alpha oscillatory activity in individuals reporting higher levels of trait anxiety.

#### 3.5.2. Resting State Alpha Oscillatory Activity Changes and Other Treatment Effects

Finally, we explored the extent to which changes in resting state alpha oscillatory activity were associated with the observed treatment effects on subjective stress and cortisol response to the cognitive stressor. Correlations indicated that the change in posterior alpha was not associated with the change in subjective stress response at one hour, nor the change in cortisol response at three hours.

**Table 5:** Resting state posterior alpha activity for MEG participants divided by trait anxiety (Mean (M) and SD).

	Low Anxiety (*n* = 8)		High Anxiety (*n* = 9)	
	M	(SD)	M	(SD)
**Placebo**	43.12	(11.94)	41.46	(11.56)
**Active**	42.95	(14.28)	45.19	(10.63)
****	N.S.		*	

* *p* < 0.05, N.S., *p* > 0.05.

## 4. Discussion

This study explored the effects of an l-theanine-based nutrient drink in response to a cognitive stressor, in addition to assessing cognitive performance and resting state alpha oscillatory activity using MEG. Using a double-blind, placebo-controlled, balanced crossover design, the primary outcome measure, subjective stress response to a cognitive stressor, was found to be significantly reduced one hour after administration of the active nutrient drink containing 200 mg of l-theanine when compared to placebo. Analysis of salivary cortisol, the primary hormone product of the hypothalamic-pituitary-adrenal axis (HPA axis) secreted in response to a stressor, revealed significantly decreased salivary cortisol response to the stressor three hours post-dose. This effect was not apparent one hour after treatment ingestion. Further, resting state alpha oscillatory activity was significantly greater in posterior MEG sensors after active treatment compared to placebo two hours post-dose; however, this effect was only apparent for those high in trait anxiety. This change in resting state alpha oscillatory activity was not correlated with the change in subjective stress response or the cortisol response, suggesting that this change in resting alpha activity was not directly related to the observed anti-stress effects of treatment administration within this sample.

Taken together with previous reports of acute anti-stress effects [19,21,22,26], the outcomes of the present study provide additional support for an anxiolytic or anti-stress effect of l-theanine administration. Studies of chronic l-theanine administration have also demonstrated anti-stress benefits, with daily doses of l-theanine (400 mg, including a one week run-in) reported to decrease subjective stress and salivary α-amylase in undergraduate students undergoing a stressful training period [63]. Furthermore, whilst not a direct study of l-theanine in isolation, Steptoe *et al.* [64] found that six weeks of black tea administration reduced platelet activation and cortisol levels in response to cognitive and behavioural stressors compared to a control matched for caffeine level. The present findings are consistent with previous reports of an anti-stress effect in response to an acute stressor [21,22], while others have reported treatment-related changes in resting mood [19,26]. The discrepancy is possibly due to the variability in laboratory stressors and measurement instruments used; however, increasing evidence supports an anti-stress effect of l-theanine administration.

It is unclear why the subjective stress and cortisol effects were observed at different post-dose time points; however, possible explanations lie in the timing of saliva sampling. Mean cortisol levels showed a downward trend across the testing day, being considerably higher at baseline assessment, consistent with the known circadian rhythm regulated by the suprachiasmatic nucleus in the hypothalamus [53]. In addition, it is likely that baseline assessment also captured meal-induced cortisol stimulation, as participants received a light standardized meal 30 min prior to baseline testing. As cortisol reduced slightly from pre- to post-stressor at the one hour post-dose assessment, it is possible that this time point captured the steeper decline associated with the meal-induced cortisol response, masking any potential stressor-related peaks and subsequent treatment-related effects. An alternative explanation for the discrepancy in timing of subjective stress and cortisol effects is the potential for a delay between HPA axis activation as a result of the stressor and changes in cortisol concentration in saliva. Cortisol measured in saliva must passively diffuse into saliva after release from the adrenal gland into the general circulation. Granger *et al.* [65] suggest that the delay for HPA axis activation being reflected in salivary cortisol concentration can be 15–20 min and in the context of acute psychosocial stress reaches peak concentrations 10–30 min after concluding the stressor [66]. The present investigation assessed saliva immediately following completion of the stressor; thus, the observed treatment effects may have captured a more cumulative effect of response to the stressor after the third completion of the stressor three hours post-dose.

The outcomes of resting state MEG recordings demonstrated increased alpha oscillatory activity across posterior brain regions after treatment with the l-theanine-based nutrient drink. Consistent with previous research [24,28,30], this effect was apparent only for those higher in trait anxiety. Trait anxiety scores in the high anxiety group ranged from 60th–90th percentile ranks based on Australian adult population norms [67]; as such, this higher anxiety group represents moderate to high levels of trait anxiety. While there is evidence to suggest anxiety disorders may be associated with reduced alpha activity during resting recordings [68], high and low anxiety groups did not differ in resting posterior alpha power at the placebo visit, suggesting that this alpha-enhancing effect was not to restore an existing deficit of posterior alpha within these non-clinical high trait anxiety individuals. Furthermore, as these changes in posterior alpha oscillatory activity were not correlated with the subjective stress response one hour post-dose or the reduced cortisol response three hours post-dose, further research is required in order to establish the functional relevance of this effect.

Whilst this investigation was framed around the l-theanine content of the active treatment, other constituents unique to the active treatment were also included, albeit at very low doses. The potential contribution of these ingredients to the observed effects cannot be discounted. A similar design, with the inclusion of a third treatment arm containing only the l-theanine content, would be required to determine the role of these additional ingredients.

## 5. Conclusions

The findings of the present study further support the anti-stress effects of l-theanine. Following administration of the active treatment, a nutrient beverage containing 200 mg of l-theanine, in addition to smaller doses of PS, alpha GPC and chamomile, subjective stress response to a cognitive stressor was found to be significantly reduced one hour post-dose, and cortisol response was significantly reduced three hours post-dose, using a double-blind, placebo-controlled, balanced crossover design. No differences in cognitive performance were observed. In higher trait anxiety participants, posterior alpha oscillatory activity was found to be increased by active treatment using resting state MEG recordings; however, this effect was not correlated with other anti-stress outcomes, suggesting further work is required to assess the functional relevance of these changes.
